# Particulate Matter-Induced Inflammation/Oxidative Stress in Macrophages: Fucosterol from *Padina boryana* as a Potent Protector, Activated via NF-κB/MAPK Pathways and Nrf2/HO-1 Involvement

**DOI:** 10.3390/md18120628

**Published:** 2020-12-09

**Authors:** Thilina U. Jayawardena, K. K. Asanka Sanjeewa, Hyo-Geun Lee, D. P. Nagahawatta, Hye-Won Yang, Min-Cheol Kang, You-Jin Jeon

**Affiliations:** 1 Department of Marine Life Sciences, Jeju National University, Jeju 690-756, Korea; tujayawardena@jejunu.ac.kr (T.U.J.); asanka@jejunu.ac.kr (K.K.A.S.); hyogeunlee92@jejunu.ac.kr (H.-G.L.); pramuditha1992@jejunu.ac.kr (D.P.N.); koty221@naver.com (H.-W.Y.); 2 Research Group of Process Engineering, Korea Food Research Institute, Jeollabuk-do 55365, Korea; 3 Marine Science Institute, Jeju National University, Jeju 63333, Korea

**Keywords:** *Padina boryana*, RAW 264.7 macrophages, Nrf2/HO-1, MAPK, NF-κB

## Abstract

Fucosterol is a phytosterol that is abundant in marine brown algae and is a renowned secondary metabolite. However, its ability to protect macrophages against particulate matter (PM) has not been clarified with regard to inflammation; thus, this study aimed to illustrate the above. *Padina boryana,* a brown algae that is widespread in Indo–Pacific waters, was applied in the isolation of fucosterol. Isolation was conducted using silica open columns, while identification was assisted with gas chromatography-mass spectroscopy (GC-MS) and NMR. Elevated levels of PM led the research objectives toward the implementation of it as a stimulant. Both inflammation and oxidative stress were caused due the fact of its effect. RAW 264.7 macrophages were used as a model system to evaluate the process. It was apparent that the increased NO production levels, due to the PM, were mediated through the inflammatory mediators, such as inducible nitric oxide synthase (iNOS), cyclooxygenase-2 (COX-2) and pro-inflammatory cytokines (i.e., interleukin-6 (IL-6), interleukin-1 (IL-1β) and tumor necrosis factor-α (TNF-α), including prostaglandin E2 (PGE_2_)). Further, investigations provided solid evidence regarding the involvement of NF-κB and mitogen-activated protein kinases (MAPKs) in the process. Oxidative stress/inflammation which are inseparable components of the cellular homeostasis were intersected through the Nrf2/HO-1 pathway. Conclusively, fucosterol is a potent protector against PM-induced inflammation in macrophages and hence be utilized as natural product secondary metabolite in a sustainable manner.

## 1. Introduction

The destruction of the ecological environment is being contributed to by various factors such as biologically hazardous and chemical waste. Over the past decade, ambient air pollution through vehicle emission dust and industrial emissions has increased. It has been reported that air pollution is the world’s single largest environmental health risk [1]. Airborne particulate matter (PM) is associated with various health risks including respiratory disorders, allergic reactions, cardiovascular diseases and dermal diseases. PM has become a major concern globally, in particular in the East Asia region including China, Korea and Japan. Beijing is considered as one of the most heaviest air polluted cities in the world [2]. Though anthropogenic sources are contributing towards this, a major natural contributor is the particulate matter originating during the spring season in the Loess Plateau, the desert regions of Mongolia and northwest China. Lee et al. (2015) reported that emissions released by China were much smaller compared with non-anthropogenic sources [3].

PM is a complex mixture of biological materials (e.g., pollen, micro-organisms), metallic ions, organic matter and poly-aromatic hydrocarbons. The inorganic composition of fine dust was characterized by Maxwell et al. (2004) using Asian dust, and the main components were reported as water-soluble mineral dust including Mg^2+^ and Ca^2+^. Further, fine-particle negative ions remained as nitrate (NO_3_^−^) and sulfate (SO_4_^2−^) associated with ammonium (NH_4_^+^) or potassium (K^+^) [4]. Factors which influence the toxicity of PM were reported by Harrison and Yin (2000) as being bulk chemical composition, trace element content, strong acid content, sulfate content and particle size distribution [5]. Lv et al. (2016) systematically analyzed PM 2.5 Beijing urban fine dust and its sources descriptively with environmental impacts [6]. Particulate matter is generally composed of coarse and fine fractions. The coarse fraction consists mainly of natural sources such as re-suspended dust and biological material (e.g., pollen, bacteria), whereas the fine particles, which are less than 2.5 µm, are dominated by anthropogenic emissions [7].

Earlier reports have indicated that the metal content and its acidity, specifically transition metals, interfere with host defense mechanisms and cause inflammation [8,9]. In vitro PM pollution studies have exhibited cytotoxicity, oxygen radical formation and cytokine release, signifying the effect of particulate pollution in inflammatory disorders [10,11]. Shukla et al. (1999) reported on the effect of fine particulate matter inhalation and nuclear factor kappa-B (NF-κB)-related inflammatory activation in pulmonary epithelial cells [12]. Similarly, Zhao et al. (2016) reported on the effect of fine dust in inflammatory pathway activation through reactive oxygen species (ROS)-dependent mechanisms [13]. ROS plays an important role in the elimination of microbes inside the lungs, though the over production of ROS results in oxidative stress due to the fact that live cells are damaged, leading to internal disorders. Respiratory diseases, excessive inflammation and oxidative stress are reported as the major causes [14,15].

During their lifespan, marine algae are exposed to extreme conditions. They face high oxygen concentrations, intense light, UV radiation and stress. Due to the fact of their rich bioactive components, stressful conditions can be overcome successfully [16]. Earlier studies focused on the different bioactive components from brown algae such as fucoxanthin [16], chromenes [17] and diphlorethohydroxycarmalol [18].

*Padina boryana* is a brown algae species that is widespread in warm Indo–Pacific waters. This species has been the subject of study on several previous occasions. Fernando et al. (2018) used the carbohydrase assisted extraction for *P. boryana* and evaluated its antioxidant and anti-inflammatory potentials briefly [19]. Fucoidan from this species has been widely studied for its structure and anticancer activity by Usoltseva et al. (2017) [20].

Fucosterol is phytosterol, apparently abundant in brown algae, and was first identified in its pure form by Heilbron et al. (1934) who published an article highlighting its potentials [21]. Its various properties have been reported vividly on different occasions. The antioxidant effects of fucosterol were reported using *Pelvetia siliquosa* [22]. Its anti-inflammatory properties against LPS-stimulated conditions were earlier reported by Jung et al. (2013) using brown algae, *Elsenia bicyclis* [23]. The anti-osteoporotic effect was evaluated using fucosterol derived from *Undaria pinnatifida* [24]. Fernando et al. (2019) studied and reported on the potential of fucosterol to inhibit particulate matter-induced inflammation and oxidative stress in the alveolar cell line A549 [25].

This study aimed to isolate fucosterol from the brown algae *P. boryana* and to evaluate its anti-inflammatory properties on PM-induced RAW 264.7 macrophages. Pure compound fucosterol identification was assisted by nuclear magnetic resonance spectroscopy (NMR) and gas chromatography-mass spectroscopy (GC-MS) data. The preliminary studies revealed its potential to inhibit PM-induced inflammation. Hence, further studies were conducted to confirm its activity using RT-qPCR techniques for gene expression analysis and Western blotting as well as enzyme-linked immunosorbent assay (ELISA) techniques. The activity was anticipated to occur via the mitogen-activated protein kinase (MAPK) and NF-κB pathways. Further, we elevated our studies to the level of oxidative stress-related protein expression analysis. To the best of our knowledge, this is the first report with regard to the assessment of fucosterol on particulate matter-induced inflammation in RAW 264.7 macrophages.

## 2. Results

### 2.1. Characterization of Particulate Matter

**Figure 1:**
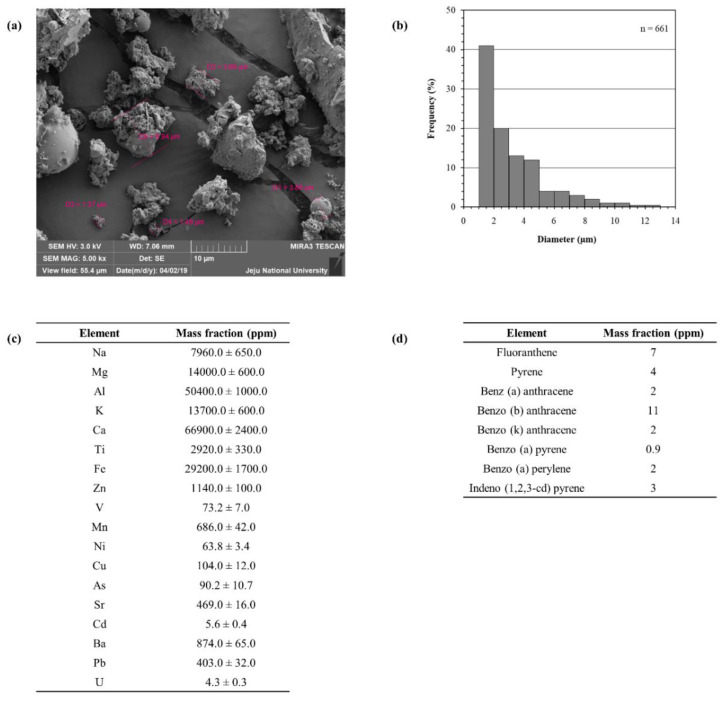
Physical and chemical parameters of particulate matter (PM) (certified reference material (CRM) No. 28). (**a**) Scanning electron microscopic (SEM) image, (**b**) distribution of particle size, (**c**) elemental composition as mass fractions and (**d**) polycyclic aromatic hydrocarbon composition. Except for the SEM image, the figures were delivered from the National Institute for Environmental Studies (NIES), Ibaraki, Japan, CRM No. 28 certificate.

Certified reference material No. 28; China fine dust particulate matter (PM) was used for the experiments. Mori et al. (2008) reported the detailed procedures for the collection of particulate matter through mechanical vibration and the chemical characterization [26]. Scanning electron microscope imaging was conducted in order to evaluate the particle size and distribution (Figure 1a). It was evident that the majority of particles possessed a diameter less than 5 µm. Furthermore, we referred to the data provided by the National Institute for Environmental Studies (NIES), Ibaraki, Japan, and supply them below in Figure 1b–d for reference. 

### 2.2. Fucosterol: Structural Analysis

The purification procedure was assisted by a bioassay guided evaluation. The sample fractions’ potential to protect macrophages against PM-stimulated inflammation and cytotoxicity was used. The chemical character was monitored via thin layer chromatography (TLC). The pure compound expressed a white colored powdered texture (Supplementary Materials, Figure S1 illustrates the purification procedure; Figure S2 depicts the GCMS analysis data for the purified compound). The molecular ion peak was observed at 412.40, and this agreed with the theoretical molecular weight of fucosterol (412.69 g mol^−1^). Further, the fragmentation pattern and relevant fragments agreed with the library spectrum of NIST 11 (the ^1^H and ^13^C data, which are provided in Supplementary Materials Figure S3, were used for the structure elucidation of fucosterol). Moreover, heteronuclear single-quantum coherence spectroscopy (HSQC) and heteronuclear multiple-bond correlation (HMBC) data assisted in confirming the carbon multiplicity and that the carbon positions correlated to protons. Previously published data were referred to in the structure’s elucidation [25,27].

### 2.3. Effect of Fucosterol (FST) against PM-Induced Cell Viability and NO Production

**Figure 2:**
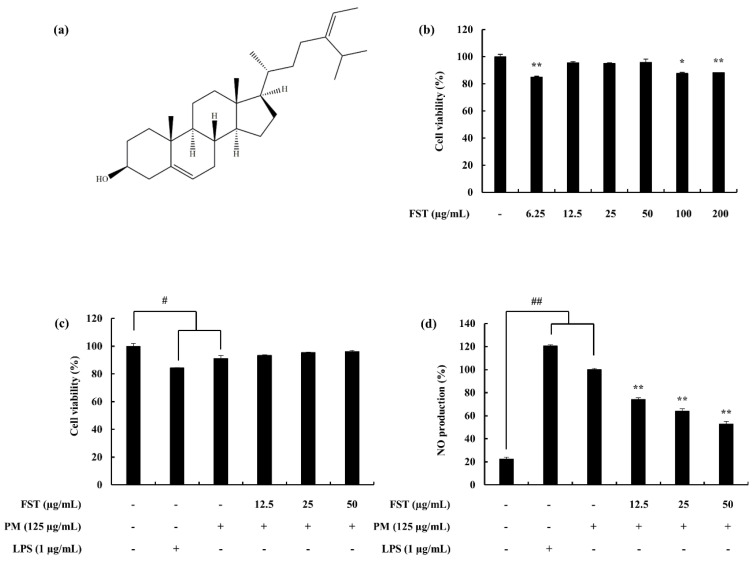
(**a**) Fucosterol (FST) skeletal formula, (**b**) cytotoxicity data for the FST sample against macrophages, (**c**) cell viability and (**d**) NO production of RAW macrophages against PM/LPS-stimulated conditions and the potential of FST to inhibit them. Cells were seeded and treated with FST after 24 h, incubated for 1 h and then co-treated with PM (125 µg/mL)/LPS (1 µg/mL). Triplicated experiments were used to evaluate the data. The results are represented as the mean ± SD. * *p* < 0.05, ** *p* < 0.01. # Denotes significance compared to the control, * represents significance compared to the PM treated group.

The protective effect of fucosterol (FST) on PM-induced macrophages are demonstrated in Figure 2. Cell viability, which was significantly affected by PM, recovered with FST exhibiting its cytoprotective effect. NO production, which was upregulated via PM, was significantly and dose-dependently downregulated through treatment with FST. LPS was used as a reference standard to compare against the data from the PM stimulation. Accordingly, LPS affected the macrophages above the level of the PM stimulation.

### 2.4. Potential of FST to Inhibit Inflammatory Mediators Driven through PM, Measured via ELISA, Western Blotting and mRNA Analysis

**Figure 3:**
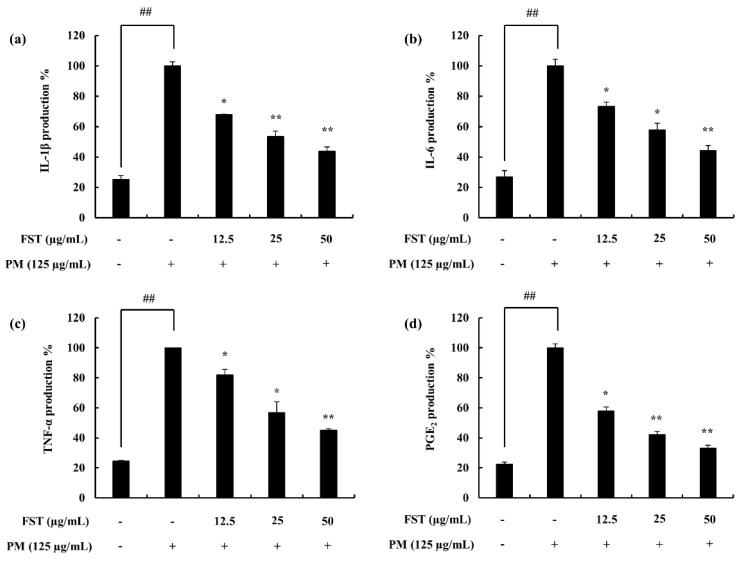
Assessing the pro-inflammatory cytokine inhibition activity of FST in PM-stimulated RAW 264.7 macrophages using enzyme-linked immunosorbent assay (ELISA) techniques. (**a**) Interleukin-1 (IL-1β), (**b**) interleukin-6 (IL-6), (**c**) tumor necrosis factor-α (TNF-α) and (**d**) prostaglandin E2 (PGE_2_). Culture supernatants of RAW 264.7 cells after successive treatment of PM were used to quantify the inflammatory cytokines and PGE_2_. Triplicated experiments were used to evaluate the data and the results are represented as the mean ± SD. * *p* < 0.05, ** *p* < 0.01. # Denotes significance compared to the control, * represents significance compared to the PM treated group.

**Figure 4:**
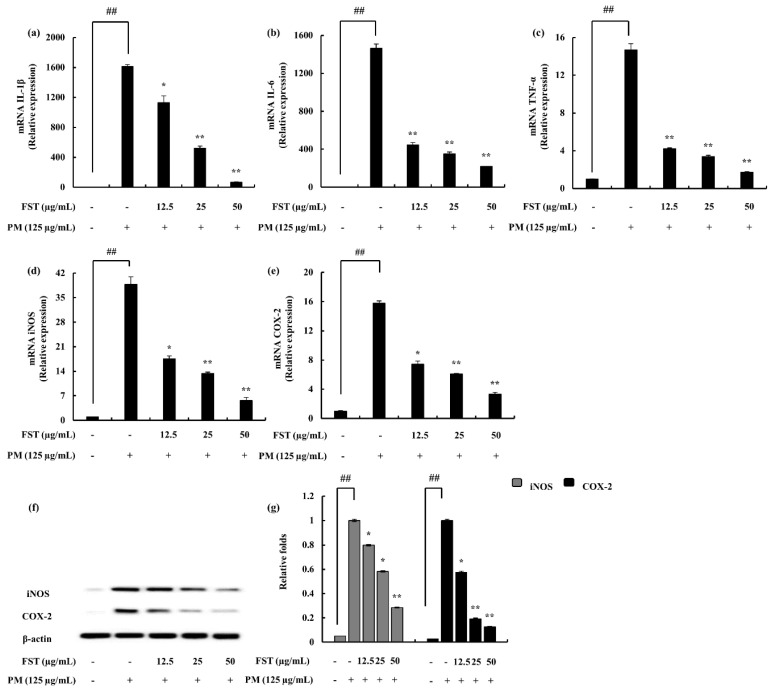
Gene expression analysis using RT-qPCR techniques. (**a**) IL-1β, (**b**) IL-6, (**c**) TNF-α, (**d**) inducible nitric oxide synthase (iNOS) and (**e**) cyclooxygenase-2 (COX-2). The 2^−ΔΔCt^ method was used to calculate the relative mRNA levels. GAPDH was used as an internal reference. Triplicated experiments were conducted. mRNA significance relative to non-treated control was calculated using the Mann–Whitney U test. * *p* < 0.05, ** *p* < 0.01. Inflammatory mediators (**f**) iNOS and COX-2 and (**g**) quantitative data were measured using Western blotting. β-actin (for cytoplasm) was used as internal control. Quantitative data was analyzed using ImageJ software. The results are expressed as the mean ± SD of three separate experiments. * *p* < 0.05, ** *p* < 0.01. # Denotes significance compared to the control while * represents significance compared to the PM-treated group.

Pro-inflammatory cytokines (i.e., interleukin-1 (IL-1β), interleukin-6 (IL-6) and tumor necrosis factor-α (TNF-α)) lead to the upregulation of inflammatory end products. It was observed to be successfully downregulated with FST treatment (Figure 3). Prostaglandin E2 (PGE_2_) correlated with the cyclooxygenase-2 (COX-2), while inducible nitric oxide synthase (iNOS) catalyzing the production of NO was downregulated. These results were evident through gene expression levels as well as the Western blot results (Figure 4).

**Figure 5:**
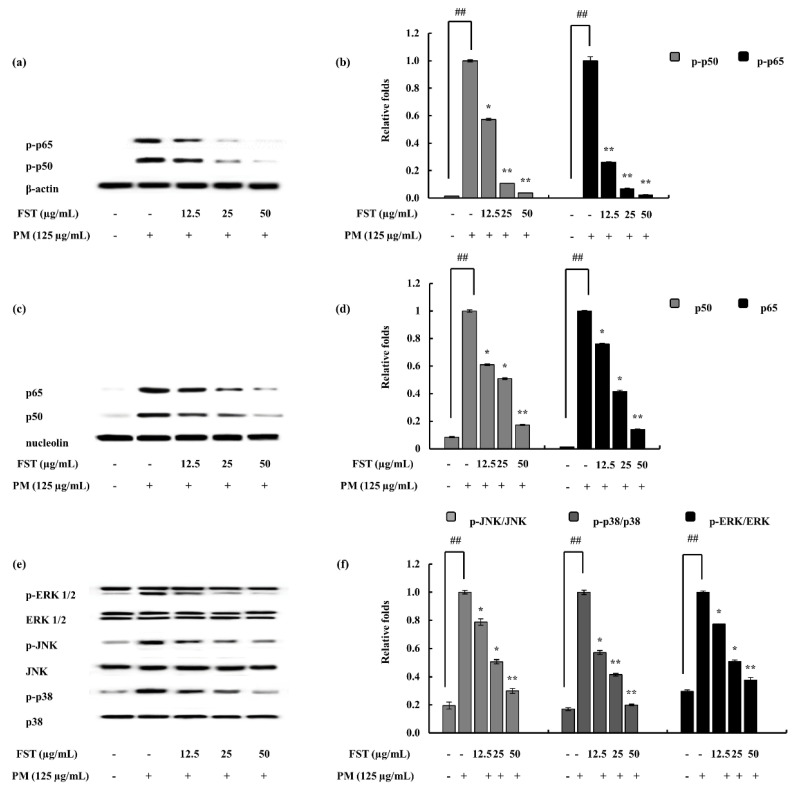
Evaluation of nuclear factor kappa-B (NF-κB) and mitogen-activated protein kinase (MAPK) pathway proteins in macrophages under PM-stimulated and FST-treated conditions. Data were obtained via Western blotting techniques and subsequent quantification of them with the use of ImageJ software. (**a**) p50 and p65 in cytosol, (**b**) quantitative data, (**c**) p50 and p65 in nucleus, (**d**) quantitative data, (**e**) p38, JNK and ERK and relevant (**f**) quantitative data. β-actin (for cytoplasm) and nucleolin (for nucleus) were used as internal controls. Results are expressed as the mean ± SD of three separate experiments. * *p* < 0.05, ** *p* < 0.01. # Denotes significance compared to the control while * represents significance compared to the PM-treated group.

The phosphorylation of transcription factors led to the production of inflammatory cytokines, hence, continuing the chain of inflammatory signaling. The results explain the decline in the phosphorylation of NF-κB signals, inferring the inhibition of inflammation through the particular pathway. Similarly, MAPK phosphorylation was downregulated through the activity of FST (Figure 5).

### 2.5. Relation to ROS via the Oxidative Stress Pathway

The combination of the antioxidant response element (ARE) and nuclear factor erythroid 2-related factor 2 (Nrf2) plays a vital role concerning the cellular defense mechanism through the activation of a wide array of antioxidants as well as detoxification components on a transcriptional level. During dormant conditions of the cell, Nrf2 is attached with Kelch-like ECH-associated protein 1 (Keap1) in the cytoplasm; under stressful conditions, Nrf2 is dissociated from the above complex and translocated to the nucleus [28].

**Figure 6:**
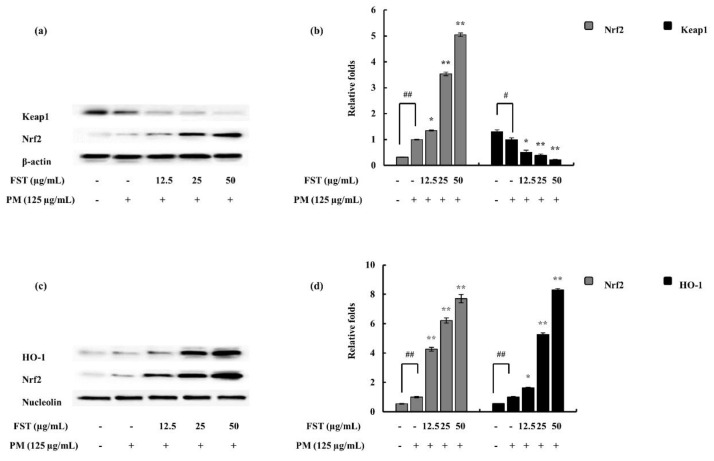
Effect of FST on PM-stimulated oxidative stress pathway-related proteins and their evaluation via Western blotting. (**a**) Nuclear factor erythroid 2-related factor 2 (Nrf2) and Kelch-like ECH-associated protein 1 (Keap1) in cytosol and their (**b**) quantitative data. (**c**) HO-1 and Nrf2 in the nucleus and respective (**d**) quantitative data. * *p* < 0.05, ** *p* < 0.01 versus the PM-treated group or # *p* < 0.05, ## *p* < 0.01 versus the un-stimulated group. β-actin (for cytoplasm) and nucleolin (for nucleus) were used as internal controls. Quantitative data were analyzed using ImageJ software. Results are expressed as the mean ± SD of three separate experiments. * *p* < 0.05, ** *p* < 0.01. # Denotes significance compared to the control while * represents significance compared to the PM-treated group.

Western blotting was implemented to observe the expression of Nrf2 and Keap1 in the presence and absence of FST under PM-stimulated conditions. It was well observed that Nrf2 expression was upregulated under PM stimulation and, with FST, significantly activated with reference to the control. The resulting antioxidant transcription factor, HO-1, was evidently upregulated in a dose-dependent manner (Figure 6).

## 3. Discussion

Air pollution can be referred to as the accumulation of diverse pollutants in the atmospheric phase of the globe, which eventually causes harm to humans and other living organisms including the natural environment [29,30]. It is reported that there is an estimated death toll above two million around the globe annually due to the direct causes of air pollution. Further, the damage is stated to effect the respiratory system [31]. The composition of PM is complex including both solid and liquid matter in varying sizes and chemical arrangements [32]. It was determined that exposure to particulate matter causes numerous health issues such as respiratory symptoms, cardiovascular diseases, lung function disputes and premature mortality [33,34].

Brown algae are widely used in the East Asia region as a food, nutraceutical and a pharmaceutical source. These include a variety of biological properties including as an antioxidant [35], anti-inflammatory [16] and antidiabetic [36]. This study selected a brown algae species, *P. boryana*, which is abundant in the Laccadive Sea, especially along the shores of Fulhadhoo Island in the Maldives. *Padina* is a calcified brown alga, and the thallus is flattened and fan shaped. The distribution of the genus is marked in temperate and tropical waters [37]. This study focused on the hexane fraction of the ethanol extract of *P. boryana*. The hexane fraction mainly contains sterols and lipids. In a majority of eukaryotic cells, sterols are present as a significant family of lipids. Sterol synthesis follows different routes and due to the fact of this reason, exhibits alterations among family classifications. Fucosterol is identified as a primary sterol in brown algae. Sanchez-Machado et al. (2004) reports that in brown seaweed fucosterol is present above 80% among sterols [38].

The results indicated a dose-dependent downregulation against PM-induced inflammation PM can easily penetrate through the respiratory tract. Human health is affected by these particles, and in due course it could result in detrimental issues, including complications in the respiratory tract and lead to allergic reactions and inflammation-caused responses in macrophage cells. As the human body consists of natural defense mechanisms, alveolar macrophages mark the first line of defense. Alveolar macrophages are involved in phagocytosis of inhaled particles. As a model system during our study, the RAW 264.7 cell line was used. Against alien challenge and tissue injury, inflammation is a constructive host defense in order to restore the structure and function of the relevant systems. Both innate and adaptive immune responses are used depending on the situation by the human body [39]. Persistent inflammation can cause non-favorable conditions such as arthritis, multiple sclerosis and inflammatory bowel disease. Anti-inflammatory agents are used in the process and act via different mechanisms. Non-steroidal anti-inflammatory drugs (NSAIDs) are frequently used as treatments, and they do not modify the pathogenesis of inflammation [40].

The cell viability which was significantly affected due to the presence of PM was successfully restored due to the treatment with FST. Inflammatory mediators, such as PGE_2_, were also inhibited by the potential of FST. The ELISA evaluation of the pro-inflammatory cytokines, such as IL-6, IL-1β and TNF-α, suggested the potent activity of FST. Similarly, iNOS and COX-2 were also observed to be downregulated. Among these, iNOS is important in the production of NO, while PGE_2_ production is influenced via COX-2. iNOS is one of the many isoforms in the family of nitric oxide synthases (NOSs). Similarly, COX-2 is also a member of the COX family of enzymes and primarily regulates the production of PGE_2_ against inflammatory conditions [41,42].

Cytokines can be defined as proteins involved in intercellular communication. These are macromolecular proteins with higher molecular weights. Soluble cytokines are abundant while some can exist both in soluble and membrane-bound forms [43]. The cytokine level measurement was conducted using numerous methods. Two major methods implied were cytokine immunoassays using protein levels (i.e., ELISA) and mRNA levels by RT-qPCR.

The NF-κB pathway’s active molecules, p50 and p65, reside in their inactive forms in the cytosol as an IκB complex. Once the stimuli is passed, the free forms of p50 and p65 translocate to the nucleus leading to the transcription of pro-inflammatory modulators [44]. The results indicate that under PM-stimulated conditions, FST downregulates phosphorylated forms in the cytosol. Nuclear p50 and p65 levels were also downregulated. The combined results suggest the potential of FST to inhibit PM-stimulated phosphorylation of the above complex, hence leading to anti-inflammatory effects. Mitogen-activated protein kinases are a family of serine/threonine protein kinases. Against external stimuli (i.e., stress signals), MAPKs support to mediate basic biological processes via regulation of the synthesis of inflammatory mediators. This makes MAPKs potential cross-points in inflammation therapeutics [45]. The activation of the MAPK pathway leads to the activation of the transcription factor NF-κB [46]. Humans possess three distinct MAP kinase cascades: extracellular signal-regulated kinases (ERK1/2), c-Jun N-terminal kinase (JNK) and p38 MAP kinase. These are activated via different MAP kinase kinases (MKKs). Among them MKK2 is responsible for the activation of ERK, while JNK is triggered via MKK 4 and 7. p38 is activated through three MKKs, namely, MKK 3, 4 and 6 [47,48]. Cell growth, differentiation besides cell death and inflammation are associated with the p38 MAP kinase pathway [49]. Earlier studies report on the potential of fucosterol to inhibit the LPS-stimulated phosphorylation of p38 MAPK and MKK 3/6. Moreover, several studies have shown the influence of p38 MAPK with regard to the activation of NF-κB [50,51]. Hence, the results of our study can be correlated with the above, such that the p38 MAPK was downregulated significantly due to the FST treatment, and the NF-κB pathway evaluation signals (i.e., p50 and p65 phosphorylation) behaved in a similar manner. However, further studies regarding MKKs are required to solidify our results in between MAPK and NF-κB.

Nrf2/HO-1, which is an evolutionary conserved mechanism, was used briefly to assess ROS involvement in the inflammatory diseases. Keap1 is an inhibitor protein, a cysteine-rich protein which is anchored to an actin cytoskeleton. It is responsible for the cytosolic sequestration of Nrf2 under physiological conditions. Keap1 promotes ubiquitination and degradation of Nrf2 under normal physiological conditions. Under stressful conditions, in which the Nrf2-dependent cellular mechanism is active (electrophiles and oxidants are rich in this stage), Nrf2 is rapidly released from Keap1. Dissociated Nrf2 is translocated to the nucleus and binds to ARE. Keap1 also receives redox information or environmental cues via its highly reactive cysteine residues, and it is referred to as the sensor of the Nrf2–Keap1 system. The dissociation of the system is a relatively rapid event. The Nrf2 half-life time is approximately 20 min [52]. The breakdown of the system leads to Keap1 stabilization. Nrf2 also increases its half-life [53]. This allows for successful nuclear translocation and cytoprotective gene transcription (HO-1) [54]. The FST treatment disrupted the Keap1/Nrf2 association while promoting its translocation to the nucleus and stabilization of Keap1 in the cytosolic environment.

The experimental results specified that the PM stimulated MAPK and NF-κB pathway mediator molecules further activating inflammatory cytokines that were significantly downregulated by FST treatment. PM air pollution has become an immense environmental and health concern not only in the East Asian region but around the globe in multiple magnitudes. Improved understanding of cellular responses related to PM stimulation would be advantageous in counteracting its detrimental effects. *P. boryana* fucosterol exhibited its effectiveness against PM-induced inflammation and related oxidative stress in RAW 264.7 macrophages as a model. The results could be utilized in the development of steroidal anti-inflammatory drugs, such as inhalers, to counteract airway inflammatory allergies. Thus, with extended in vivo scale studies, particulate matter airway complications relief can be expected.

## 4. Materials and Methods

### 4.1. Materials

China fine dust particulate matter (PM) (certified reference material No. 28) was purchased from the National Institute for Environmental Studies, Ibaraki, Japan. The organic solvents used in the experiments were of HPLC grade and were purchased from Sigma-Aldrich (St Louis, MO, USA). Silica gel 60 F254 TLC plates were purchased from Merck (Darmstadt, Germany). Silica (30–60 mesh), for open column preparation, was purchased from Sigma-Aldrich. Deuterated chloroform for NMR analysis was obtained from Cambridge Isotope Laboratories (Andover, MA, USA). The RAW 264.7 macrophage cell line was purchased from the Korean Cell Line Bank (KCLB, Seoul, Korea). Dulbecco’s modified Eagle’s medium (DMEM) with fetal bovine serum (FBS) and antibiotics (i.e., penicillin and streptomycin) purchased from GIBCO Inc. (Grand Island, NY, USA) were used as growth medium. 3-(4,5-Dimethylthiazol-2-yl)-2,5-diphenyltetrazolium bromide (MTT) was purchased from Sigma-Aldrich. The cytokine kits used in the experiments were purchased from eBioscience (San Diego, CA, USA), R&D Systems (Minneapolis, MN, USA), BD Opteia (San Diego, CA, USA) and Invitrogen (Carlsbad, CA, USA). Santa Cruz Biotechnology (Santa Cruz, CA, USA) purchased antibodies were used for Western blotting.

### 4.2. Fucosterol

#### 4.2.1. Isolation of Fucosterol from *P. boryana*

*P. boryana* samples were collected from the Fulhadhoo Island coastal area of the Maldives in January 2018. Samples were immediately washed with running water to remove epiphytes and sand. The samples were then freeze-dried and powdered. Sample repositories were stored in the Marine Bio Resource Technology Lab, Jeju National University. Sample extraction was conducted successfully with 70% ethanol four times. This was evaporated to obtain the crude ethanolic extract of *P. boryana* (PBE). PBE was dissolved in deionized water and a stepwise fractionation was conducted (hexane, chloroform and ethyl acetate). The hexane fraction was further resolved (PBEH1–PBEH5). The elution solvent consisted of hexane and ethyl acetate in increasing polarity (9:1→7:3→1:1)–›ethyl acetate and ethyl acetate:methanol (1:1). A second silica open column was used to resolve the fraction PBEH2. The elution was done in the same solvent system with increasing polarity (85:15→65:35→1:1 and ethyl acetate). This resulted in four fractions (PBEH21–PBEH24). The PBEH22 was resolved, resulting a collection of 96 tubes. This was analyzed by TLC, and the tubes were pooled into four fractions (F1–F4). Fraction F2 was resolved via preparative TLC resulting in 7 fractions (F2A–F2G). Among these fractions, F2F indicated the presence of fucosterol (FST) and was identified as the active metabolite (Supplementary Materials Figure S1 details the purification and isolation procedures.) The sample was used for cell culture bioassays after dissolving in DMSO and successful serial dilution in culture media. The sample DMSO concentrations in working samples were maintained at less than 0.1% [25].

#### 4.2.2. Structural Characterization

GC-MS assisted in the structural confirmation (Shimadzu GCMS-TQ8040, Shimadzu Corp., Kyoto, Japan). The method involved a fused silica capillary column (RTx-5MS, 30 m × 0.25 mm i.d., 0.25 µm film thickness), an injection temperature of 280 °C and was injected via splitless mode. The oven program was 260 °C, 3 min, 6 °C/min to 320 °C, 5 °C/min to 330 °C, 2 min. The ion source temperature was maintained at 200 °C. The scan range was 50–500 m/z. Helium was used as the carrier gas with a constant flow rate of 0.73 mL/min [55].

Nuclear magnetic resonance spectroscopy (NMR) was conducted using a 400 MHz spectrometer (JNM-ECX400, JEOL, Japan). ^1^H and ^13^C NMR spectra were successfully obtained. The sample was prepared by dissolving a minute amount in CDCl_3_. The chemical shifts were demonstrated in ppm and the coupling constants in Hz. Multiplicity abbreviations were used as the following: s = singlet, d = doublet, t = triplet, dt = doublet of triplet, dd = doublet of doublet and m = multiplet. Heteronuclear single-quantum coherence (HSQC) and heteronuclear multiple-bond correlation (HMBC) were conducted to confirm the carbon multiplicity and carbon positions correlated to protons [25,27].

### 4.3. Particulate Matter: Morphological Analysis

The sample was platinum sputter coated (Quorum Technologies, Lewes, UK). The surface morphology of the CRM No. 28 particles was observed using a JSM-6700F field-emission scanning electron microscope (JEOL, Tokyo, Japan). The instrument was operated at 10.0 kV.

### 4.4. Cell Culture

#### 4.4.1. Maintenance of Cell Line

The RAW 264.7 macrophage cell line was maintained in the DMEM growth medium (10% FBS and 1% antibiotics). The cells were maintained under controlled conditions: 5% CO_2_ and 37 °C. Cells were periodically sub-cultured and used for experiments in the exponential growth phase.

#### 4.4.2. Measurement of Cell Viability

Initially, the optimum FST concentration values were determined via screening a wide range. This was followed by the evaluation of the cytoprotective effect of FST against PM/LPS-stimulated macrophages. The cells were seeded with a cell concentration of 1 × 10^5^ cells/mL (96 well plates). FST was treated after a 24 h incubation period. PM (125 µg/mL)/LPS (1 µg/mL) was treated after 1 h and continued its incubation further for 23 h. An MTT assay was performed [56]. The assay results were obtained in the 540 nm optical density value.

#### 4.4.3. Evaluation of NO Inhibition Activity

The macrophage cell line was seeded in 24 well plates. The samples were treated and were incubated for 1 h and then stimulated with PM (125 µg/mL)/LPS (1 µg/mL). At 23 h, further incubation was continued. NO production analysis was conducted using the Griess assay [57,58].

### 4.5. Assessing PGE_2_ and pro-Inflammatory Cytokine Production

Initially, the cells were seeded (24 well plates), and within 24 h the FST sample was treated. The PM (125 µg/mL) stimulation was conducted after 1 h and further incubated for 23 h. The cell media were retrieved individually and assessed for each parameter of cytokine. The manufacturer’s instructions were followed in the process.

### 4.6. FST Downregulated NF-κB and MAPK Pathway Proteins

Inflammatory mediators, such as iNOS and COX-2, including the NF-κB pathway (i.e., p50 and p65) and MAPKs (i.e., p38, ERK and JNK), were assessed via Western blotting. Further, selected oxidative stress pathway proteins were also evaluated (i.e., Nrf2, Keap1 and HO-1). Cells were seeded (six well plates) and treated with samples and induced with PM. The cells were harvested, lysed and the proteins were measured using the BCA protein assay method. The harvesting time was dependent on the type of protein analysis and followed the method explained by Jayawardena et al. (2019) [42]. Proteins were subjected to electrophoresis (sodium sulfate–polyacrylamide gels, 12%), transferred to nitrocellulose membranes and blocked with skimmed milk. Primary and secondary antibody incubation was conducted, and the bands were ultimately developed and photographed (FUSION Solo Vilber Lourmat system). The ImageJ program was used for the band intensity quantification process [58,59].

### 4.7. Gene Expression Analysis

#### 4.7.1. Extraction of RNA and cDNA Synthesis

The total RNA from the cells were extracted via a commercial extraction kit following the manufacturer’s instructions (Tri-Reagent™, Sigma-Aldrich, St. Louis, MO, USA). Depending on the quantified RNA content measured (μDrop Plate, Thermo Scientific, Waltham, MA, USA), first strand cDNA was synthesized and stored at −80 °C (prime Script™, Takara Bio Inc., Kusatsu, Shiga, Japan).

#### 4.7.2. Quantitative Real-Time PCR (qPCR) Analysis

SYBR green quantitative real-time PCR techniques were implemented to measure gene expression levels. A Thermal Cycler Dice-Real Time System (Takara, Japan) was used to complete the process. GAPDH was the internal reference standard. The experimental method followed the process explained by Jayawardena et al. (2019) [42]. Data were analyzed using the method explained by Livak and Schmittgen (2001) [60].
GAPDH, antisense; 5′-AAGGGTCATCATCTCTGCCC-3′ and
sense, 5′-GTGATGGCATGGACTGTGGT -3′;IL-1β, antisense; 5′-CAGGATGAGGACATGAGCACC-3′ and
sense, 5′-CTCTGCAGACTCAAACTCCAC -3′;IL-6, antisense; 5′-GTACTCCAGAAGACCAGAGG -3′ and
sense, 5′-TGCTGGTGACAACCACGGCC-3′;TNF-α, antisense; 5′-TTGACCTCAGCGCTGAGTTG -3′ and
sense, 5′-CCTGTAGCCCACGTCGTAGC -3′;iNOS, antisense; 5′-ATGTCCGAAGCAAACATCAC-3′ and
sense, 5′-TAATGTCCAGGAAGTAGGTG-3′;COX2, antisense; 5′-CAGCAAATCCTTGCTGTTCC -3′ and
sense, 5′-TGGGCAAAGAATGCAAACATC-3′.

### 4.8. Statistical Analysis

The experiments were conducted in triplicate and data are expressed as the mean ± standard deviation. Data were analyzed using IBM SPSS with one-way ANOVA. * *p* < 0.05, * *p* < 0.01 versus the PM-treated group or ^#^ *p* < 0.05, ^##^ *p* < 0.01 versus the un-stimulated group were considered statistically significant.

## 5. Conclusions

Fucosterol purified from *P. boryana* exhibited an effective potential against PM-induced inflammatory conditions in RAW 264.7 macrophages. NO, the distinct end product of inflammation, was successfully inhibited via fucosterol under PM-stimulated conditions. Inflammatory mediators, such as iNOS, COX-2 and PGE_2_ as well as pro-inflammatory cytokines (i.e., IL-1β, IL-6 and TNF-α), were observed to be downregulated dose-dependently with the treatment of FST. These results were further reinforced via the MAPK and NF-κB pathway signal molecule expression subdual. The Nrf2/HO-1 pathway results suggest ROS downregulation due to the activity of fucosterol. Thus, fucosterol could function as a potent protector against PM-induced inflammatory diseases. In conclusion, this study provides an understanding of PM-stimulated cellular responses and mechanisms. Further, in vivo studies would be beneficial to understand the detailed mechanisms and bioavailability of fucosterol on target organs.

## Supplementary Materials

The following are available online at https://www.mdpi.com/1660-3397/18/12/628/s1, Figure S1. Flow diagram representing the extraction and fractionation of *Padina boryana* (PC) 70% ethanol extract (PBE). Figure S2. GC-MS/MS analysis of fucosterol. Figure S3. ^1^H and ^13^C NMR spectrum of fucosterol.
