# Supplementary material for: Particulate Matter-Induced Inflammation/Oxidative Stress in Macrophages: Fucosterol from Padina boryana as a Potent Protector, Activated via NF-κB/MAPK Pathways and Nrf2/HO-1 Involvement
Source: Mar Drugs. 2020 Dec 9;18(12):628. doi: 10.3390/md18120628 (PMC7763233; doi:10.3390/md18120628)
Supplement: Supplementary file 1 [file marinedrugs-18-00628-s001.pdf]

Supplementary material

Particulate matter induced inflammation/oxidative stress in macrophages; Fucosterol from Padina boryana as a potent protector, activates via NF-κB, MAPK pathways and Nrf2/HO-1 involvement

Thilina U. Jayawardena [1], K. K. Asanka Sanjeewa [1], Hyo Geun Lee [1], D.P. Nagahawatta [1], Hye-Won Yang [1], Min-Cheol Kang [2*] and You-Jin Jeon [1, 3*]

[1] Department of Marine Life Sciences, Jeju National University, Jeju 690-756, Republic of Korea.
[2] Research Group of Process Engineering, Korea Food Research Institute, Jeollabuk-do 55365, Republic of Korea.
[3] Marine Science Institute, Jeju National University, Jeju Self-Governing Province 63333, Republic of Korea.

* Co-correspondence: **Min-Cheol Kang** (Ph.D.) networksun@naver.com; **You-Jin Jeon** (Ph.D.) youjin2014@gmail.com; Tel.: +82-064-754-3475

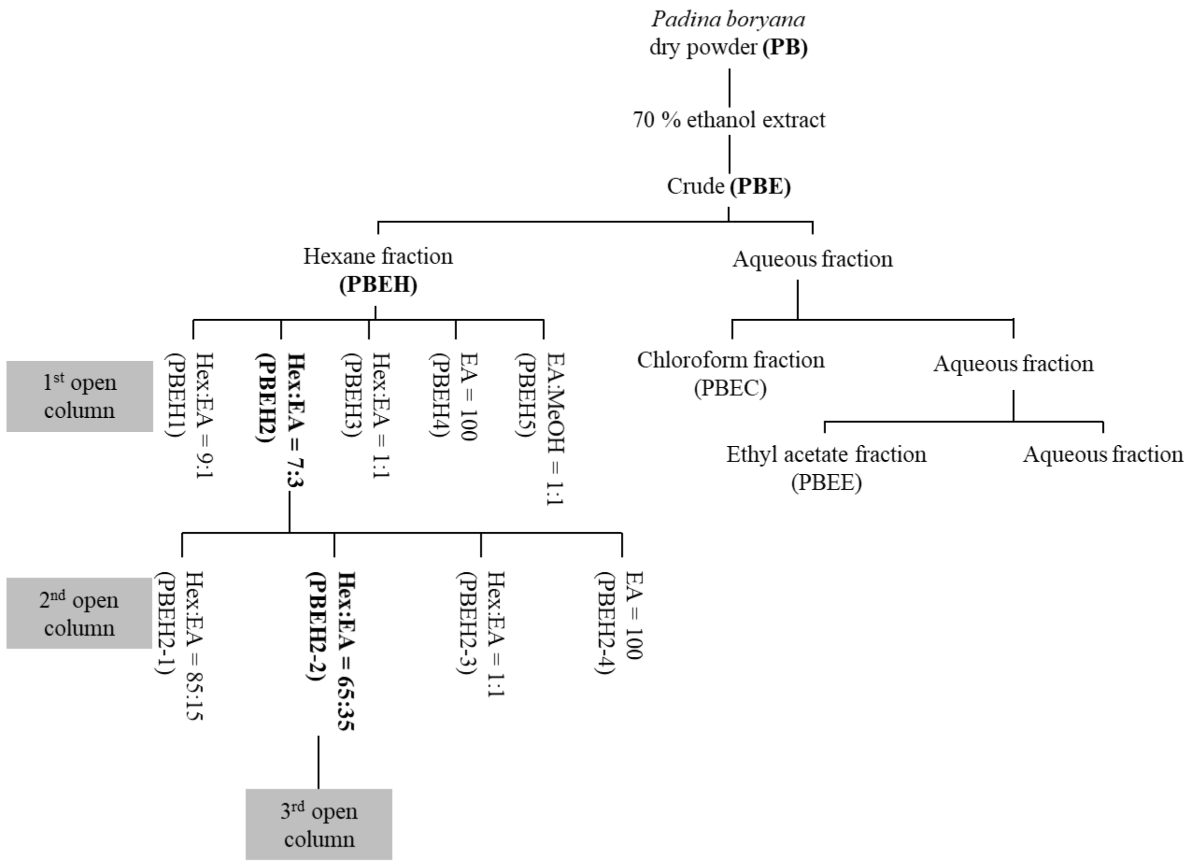

Figure S1. Flow diagram representing the extraction and fractionation of *Padina boryana* (PC) 70% ethanol extract (PBE).

Crude extract of PBE was homogenized in deionized water (DW) and fractionated between organic solvents and aqueous layer (DW) using hexane, chloroform and ethyl acetate respectively. Followed by bioassays the active hexane fraction (PBEH) was separated into 5 fractions using silica open column by eluting with 5 different solvent systems of hexane and ethyl acetate in order of increasing polarity. The bioactive second column fraction (PBEH2) was further separated into 4 fractions by a similar chromatographic method. Bioactive second column fraction (PBEH22) was selected for further purification. Each above column eluting solvent system was selected based on its separation profile as evaluated by extensive TLC analysis.

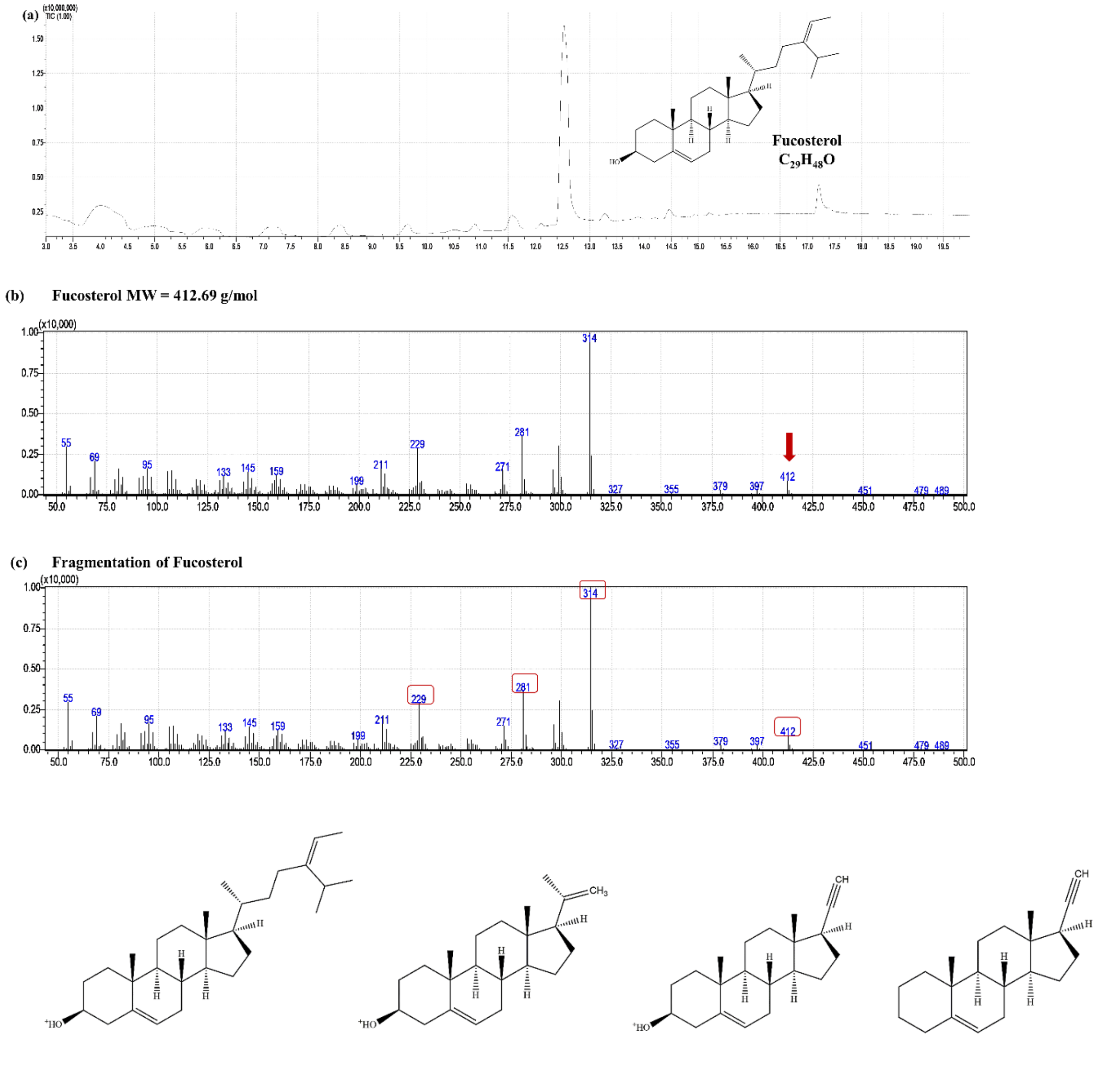

412 314 299 281

Ion mass (m/z)	Fragment
412	$[M]^+$
397	$[M-CH_3]^+$
379	$[M-CH_3-H_2O]^+$
314	$[M-C_6H_{11}-CH_3]^+$
299	$[M-C_6H_{11}-2CH_3]^+$
281	$[M-C_6H_{11}-2CH_3-H_2O]^+$
229	$[M\text{-sidechain-ring D cleavage-}CH_3\text{-}2H]^+$
213	$[M\text{-sidechain-ring D cleavage-}CH_3\text{-}H_2O]^+$

Figure S2. GC-MS/MS analysis of fucosterol.

Pure compound fucosterol was analysed by GC-MS/MS to confirm the purity and to obtain the mass fragmentation patterns. Predicted mass fragments of fucosterol corresponding to each prominent peak in the MS spectrum is presented.

(a)

(b)

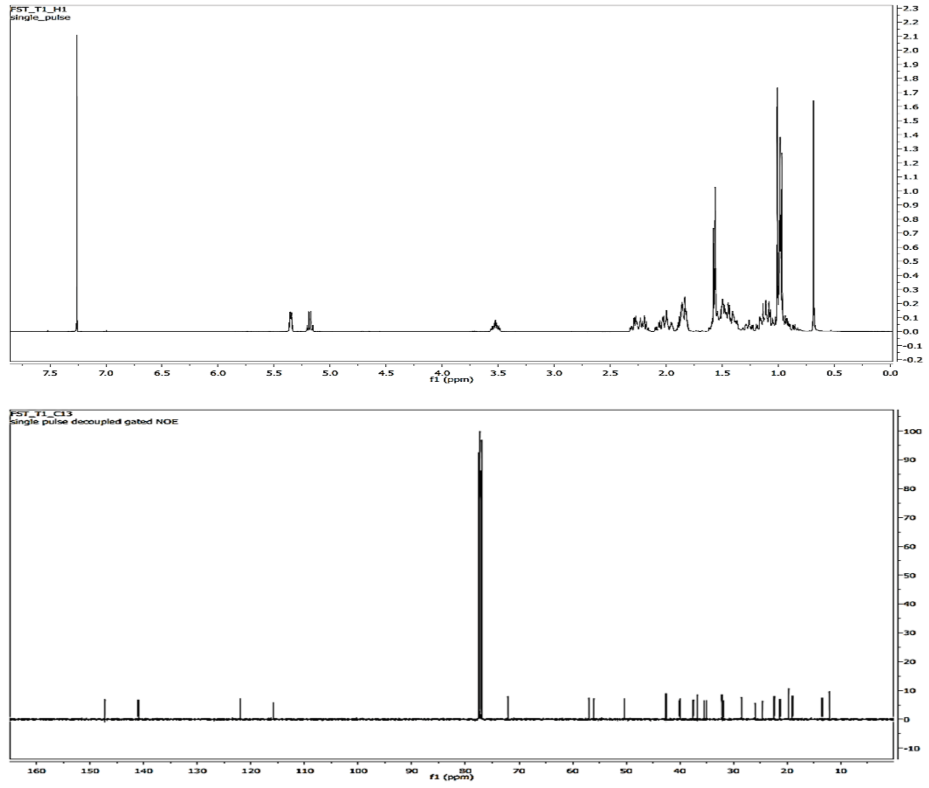

(c)

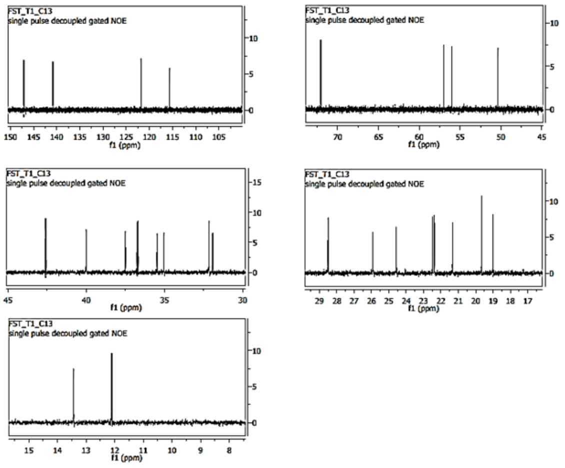

(d)

Selected ^{1}H-NMR data δ (Multiplicity/Hz)	Selected ^{13}C-NMR data δ
0.68 (s, H_{18}), 0.98 (d, 6.4, H_{21}), 1.01, 1.02 (d, 6.8, H_{26}, H_{27}), 1.00 (s, H_{19}), 1.57 (d, 7.2, H_{29}), 3.52 (m, H_3), 5.18 (q, 6.7, H_{28}), 5.35 (d, 5.2, H_6)	12.07 (C_{18}), 13.44 (C_{29}), 18.98 (C_{21}), 19.63 (C_{19}), 21.33 (C_{27}), 22.38 (C_{26}), 72.11 (C_3), 115.52 (C_{28}), 121.77 (C_6), 140.65 (C_5), 146.86 (C_{24})

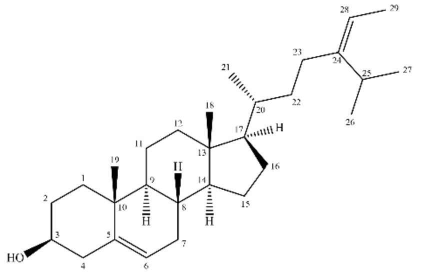

Figure S3. ^{1}H and ^{13}C NMR spectrum of fucosterol.

Pure compound the characterized using (a) ^{1}H NMR and (b) ^{13}C NMR. Sample was dissolved in deuterated chloroform for the NMR analysis. (c) Some expanded regions of ^{13}C NMR and (d) selected chemical shifts of the spectrum.
